# Implications of sleep loss or sleep deprivation on muscle strength: a systematic review

**DOI:** 10.1007/s11325-025-03413-0

**Published:** 2025-07-15

**Authors:** Judy Easow, Tulasiram Bommasamudram, Madhavi Munnilari, Ragini Adhikari, Ben J. Edwards, Kirtana Raghurama Nayak, K. Vaishali, Aishwarya Ravindrakumar, Chloe Gallagher, Samuel A. Pullinger

**Affiliations:** 1 Department of Exercise and Sports Science, Manipal College of Health Professions, Manipal Academy of Higher Education, Karnataka 576104 Manipal, India; 2 Institute for Physical Activity and Nutrition (IPAN), School of Exercise and Nutrition Sciences, Deakin University, Geelong, Australia; 3 Sport Science Department, Inspire Institute of Sport, Vidyanagar, Dist. Bellary, Karnataka India; 4 Research Institute for Sport and Exercise Sciences, Liverpool John Moores University, Liverpool, UK; 5 Department of Physiology, Kasturba Medical College, Manipal Academy of Higher Education, 576104 Manipal, India; 6 Department of Physiotherapy, Manipal College of Health Professions, Manipal Academy of Higher Education, Karnataka 576104 Manipal, India

**Keywords:** Sleep, Sleep deprivation, Muscle strength, Review, ROB, ROBINS-I

## Abstract

**Purpose:**

The aim of this review was to evaluate the current evidence regarding the impact of sleep deprivation (SD) on strength performance.

**Methods:**

An English-language literature search revealed 514 articles through primary database searches, and 563 via organization searches/citation searching. The inclusion criteria were met by thirteen articles which were included in the review. The inclusion criteria set were healthy male and/or female adult participants, a sleep loss condition (e.g. SD or partial sleep deprivation), strength measures (e.g. back strength or leg strength or grip strength), and a peer-reviewed academic paper counterbalanced and/or or randomised in design.

**Results:**

The systematic review identified a total of 13 studies that met the inclusion criteria. Some studies reported a negative impact of SD on strength performance, while others showed no significant differences following a night of SD. Both acute and chronic SD were found to result in some measures of muscle strength, power output, and muscular endurance to be significantly reduced. Additionally, impaired neuromuscular function and increased fatigue were commonly observed in sleep-deprived individuals.

**Conclusion:**

The findings of this systematic review highlight the detrimental effects of SD on strength performance. Individuals involved in strength-based activities should prioritize adequate sleep to optimize their physical performance. Further research is needed to explore the underlying mechanisms linking SD and strength impairment, as well as to investigate potential interventions to mitigate these effects. Sleep hygiene education and interventions targeting sleep duration and quality should be implemented to promote optimal strength and performance outcomes. Future research should focus on understanding the mechanisms behind these effects and developing effective interventions to mitigate the adverse consequences of SD on muscle function.

**Supplementary Information:**

The online version contains supplementary material available at 10.1007/s11325-025-03413-0.

## Introduction

Sleep plays an important role in several brain functions, and both the American Academy of Sleep Medicine and the Sleep Research Society recommend that the average adult should achieve 7–9 h of sleep per night to sustain health [1]. Partial sleep loss or sleep deprivation (SD) is classed as a reduction of sleep per night, compared to that habitually taken in a 24-hour period. SD or partial sleep deprivation (PSD) are common occurrences within our society, due to irregular work schedules, social/family obligations, and travel, resulting in 45% of the western population failing to obtain the recommended 7–9 h per night [2–4]. Poor sleep quality and duration of sleep is of an even higher prevalence in athletes due to constraints around time-zone transitions, environmental factors, demands of training and competition, psychological issues, and disturbed circadian rhythms [5–7]. A recent consensus statement has shown that habitual sleep durations in athletes are ≥ 7 h, with ≤ 50% or more exeriencing sleep disturbance, and ∼ 25% suffering ighly disturbed sleep. In recent years, there has been a rapid growth and large focus placed on sleep science as part of an advanced training and recovery plan in elite athletes. Sleep has long been recognised as a key component of recuperation and preparation for high-intensity training and performance [8].

The circadian rhythm (sleep-wake cycle) regulates alertness and sleepiness, with disturbances affecting sleep duration and sleep quality [9]. Cumulative sleep loss has shown to have major detrimental effects on the nutritional, physiological, and endocrine health of an athlete, with the consequences on performance and cognitive function extensively investigated [10, 11]. The effects of sleep loss on sport specific physical performance have been investigated less extensively [12], and findings are contradictory. When an individual is subjected to total SD for a period of one night, measures of grip strength, leg press and back strength have shown to be unaffected [4]. However, when individuals were subjected to 64-hour of SD, vertical jump performance and isokinetic knee extension strength decreased, but no differences were observed in isometric strength or 40-m dash performance [13]. A study performed by Reilly and Deykin [14] found that measures of grip strength were only reduced on the third night when partially restricted sleep was implemented for three consecutive nights, suggesting that impairments in strength are more marked following several nights of reduced sleep. It has been suggested that sleep loss affects larger muscle groups [5] and the type and demand of task affect the extent the of drop in strength performance [15].

It is believed that athletes who do more physically demanding exercises, such as clean and jerk or the snatch, will experience a higher performance drop due to SD [15]. When individuals were subjected to only 3-hour sleep for 3 consecutive nights both maximal strength and submaximal lift capacity were significantly reduced during bench press, leg press and deadlift [16]. Previous research and previous reviews have drawn attention to the fact that SD/PSD can lead to a significant reduction in muscle strength. These findings underscore the vital importance of adequate and restorative sleep for individuals, especially those who engage in regular resistance training and muscle-building exercises. Therefore, the aim of this article was to investigate at the following research question “Does sleep loss affect physical performance related to strength”?

## Methodology

### Reporting standard

The Preferred Reporting Items for Systematic Reviews and Meta-Analyses 2020 (PRISMA 2020) guidelines [17] have been followed for this systematic review. Appendix 1 presents the PRISMA 2020 checklist, providing the appropriate page numbers where items of information are found in the current manuscript.

### Eligibility criteria

The criteria for inclusion were based on the Cochrane guidelines for conducting systematic reviews [18]. All eight authors set up and agreed the inclusion and exclusion criteria for the review. Three authors (AR, JE & TB) finalised the eligibility assessment in a blinded standardised way by screening the titles and abstracts independently, after completion of the initial assessment and selection process of all studies. Accepted manuscripts had to meet the following criteria for eligibility:

1. Population– healthy male and/or female adult participants (18 to 30 yrs of age; this age group has shown to be more vulnerable to sleep loss and circadian disturbances) only.
2. Strength performance variables– such as back strength, leg strength, grip strength, intermittent sprint performance, maximal voluntary isometric contraction, maximal voluntary force, and/or voluntary activation.
3. Sleep loss condition– such as sleep loss, sleep deprivation, partial sleep deprivation (participants taking sleep medications or not having recognised sleep loss are excluded from the search due to implications on strength performance).
4. Design– Trials counterbalanced and/or randomised in design.

### Literature search strategy and information sources

**Fig. 1:**
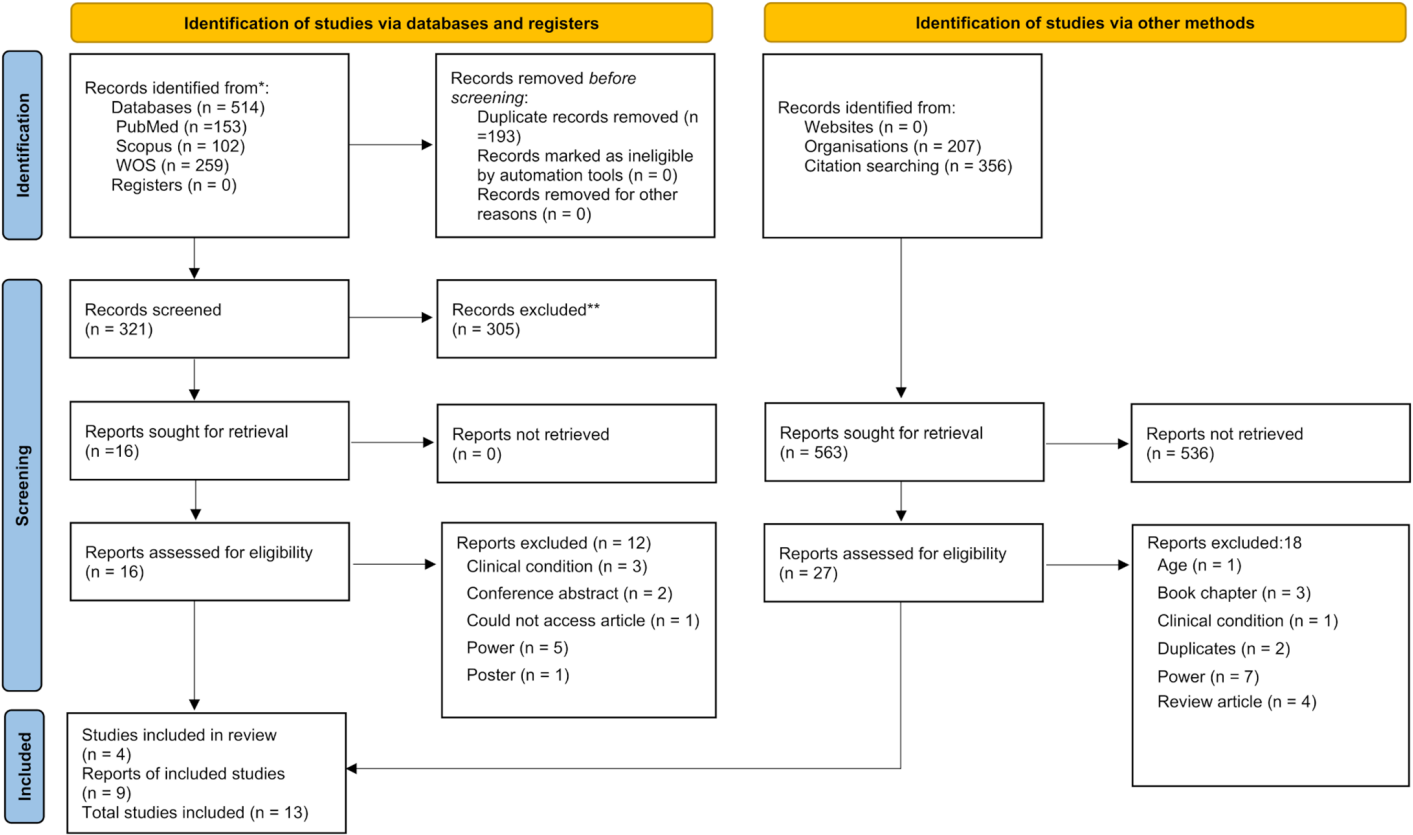
PRISMA 2020 flow diagram for new systematic reviews which included searches of databases, registers and other sources

An in-depth computerised literature search of the grey literature in the English-language was performed by three authors (CG, SP & TB). This was done through Liverpool John Moores University electronic library, Manipal Academy of Higher Education electronic library; and three electronic databases: PubMed (MEDLINE), Scopus, and Web of Science (August 2023– December 2023) and was completed on the 12th of December 2023. A search for relevant content related to SD variables and time-of-day variation using the following search syntax using Boolean operators in titles, abstracts, and keywords of indexed documents: (“sleep loss” OR “sleep deprivation” OR “total sleep deprivation” OR “sleep restriction” OR “insomnia” OR “sleep disruption” OR “inadequate sleep” OR “micro-sleep” OR “insufficient sleep” OR “sleep apnoea”) AND (“muscular strength” OR “muscle” OR “power” OR “hypertrophy” OR “muscle growth” OR “performance”). Additional advanced search techniques using wildcards, truncation and proximity searching were incorporated. Two authors (JE & TB) performed the secondary searches by screening the reference list of all manuscripts included in the review to search for additional papers meeting inclusion criteria. To explore further follow-up studies, forward reference searching was done through authors and citations. To reduce potential selection bias, one author (SP) carried out the searches for study selection independently. However, our study relied on published literature without consulting external experts or searching for unpublished studies. The study selection process with detailed information on the flow of papers including searches of databases, registers and other sources is presented in Fig. 1.

### Study selection

The article was included in the review if male and/or female participants were identified as the study population. When the abstract and/or title did not provide sufficient information or detail around the eligibility and relevance of the article for the review, one of the authors (SP) read the full article. By doing so, it was possible to determine whether the article met the required inclusion criteria. If the main purpose of the article was not a study looking at the effect of sleep loss/deprivation on muscle strength performance, the manuscript was not included in the review. Any literature reviews, conference abstracts and letters to the editor were not deemed eligible for the review as studies such as these are not methodologically-quality-assessable and/or critically appraisable.

### Data extraction

Three authors (AR, CG, and JE) performed the data extraction independently and a final data check was done by a fourth author (SP). Studies included in the review had the following extraction of data: (1) the authors and year of publication; (2) the total number of participants and their characteristics (e.g., age, body mass and stature); (3) retiring and rising times; (4) type of sleep condition (e.g., normal sleep, PSD, 24-hour SD); (5) the muscle strength test(s) performed and equipment used; (6) the strength performance variables assessed (e.g., grip strength, bench press, clean and jerk, maximal isometric and isokinetic strength); (7) the significance established with *P* values; and (8) % difference and values for strength measures between conditions (if in-depth results were provided).

### Quality assessment

**Fig. 2:**
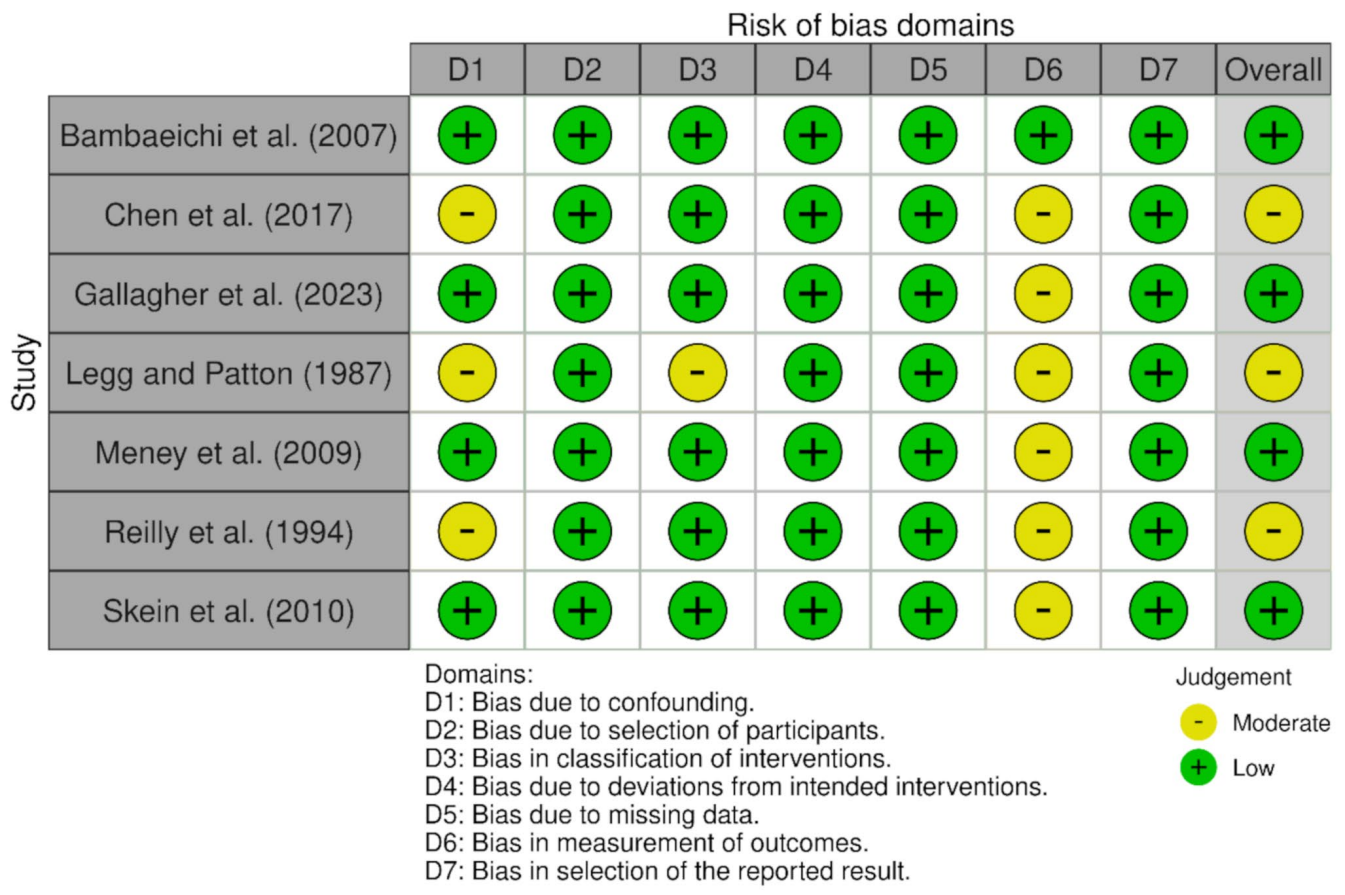
Risk of bias of the seven included studies, according to the ROBINS-I tool using the “traffic light” plots of the domain-level judgements for each individual result (McGuinnes & Higgins, 2020)

**Fig. 3:**
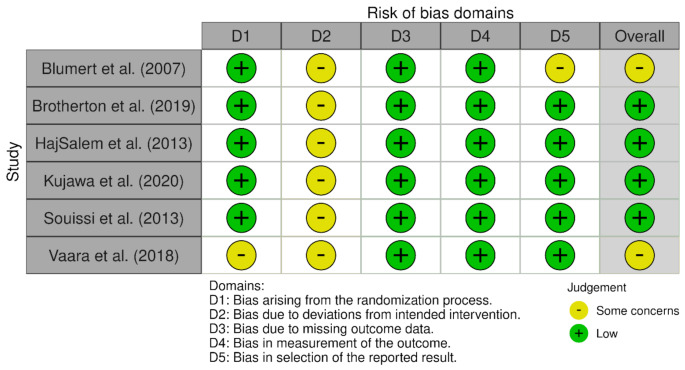
Risk of bias of the six included studies, according to the RoB 2.0 tool using the “traffic light” plots of the domain-level judgements for each individual result (McGuinnes & Higgins, 2020)

To evaluate the risk of bias in the study, two distinct tools were employed, following the Cochrane Scientific Committee’s quality assessment recommendations. Studies which were randomised were assessed using the Risk of Bias (ROB) 2.0 tool, while non-randomised were assessed using the ROBINS-I tool. The tools focus on identifiable results that are structured according to specific sets of domains of bias, that include signalling questions that provide information related to risk of bias judgements, leading to an overall risk of bias judgement. The tools are then scored and further broken down into risk of bias ranging from “low” to “moderate” to “serious” to “critical”. Two reviewers (JE and CG) evaluated the risk of bias for all articles independently and observed disagreement in only 6 domains of risk of bias over the 13 studies (6.9%). A third reviewer (SP) resolved the differences observed. Figures 2 and 3 provide a visualisation of the results using “traffic light” plots of the domain-level judgements for each individual result using the guidelines of each tool of evaluation.

## Results

### Search results

The search of the literature revealed a total of 514 articles through the primary database search. A further 563 articles were acquired through citation and organisation (University databases) searches. The number of articles discovered in electronic database searches or other methods used can be found in a detailed flow chart of the literature in Table 1. Following the removal of duplicate articles, a total of 321 titles acquired via databases remained in the reference manager (Mendeley, Elsevier, Amsterdam, The Netherlands). All titles, abstracts, and keywords were examined, and 16 articles remained and were assessed for inclusion eligibility. From these, 4 met the criteria and were included within this systematic review. A further 27 articles were identified via citation and organisation searching and assessed for inclusion eligibility. Nine articles met the inclusion criteria set, taking the number of accepted manuscripts to thirteen. Figure 1 provides the reasons for exclusion of articles in detail.

**Table 1:** Summary of the articles reviewed for sleep deprivation and muscle strength (*n* = 13) with an overview of the participants, the experimental protocols with sleep deprivation, strength test, the variables examined, and the main findings related to sleep deprivation and muscle strength in relation to each variable

Author and Date	Participants	Retiring and rising times	Sleep Conditions	Strength Test	Performance variables examined	Significance of main effects between conditions	Main findings
Bambaeichi et al. (2007)	8 sedentary eumenorrheic females	Retiring: 22:30 − 23:30 h: m Rising: 05:30 h: m	N	Muscle strength of knee (Isokinetic dynamomter - Lido Active, Loredan, Davis, CA, USA)	Maximal isometric and isokinetic knee strength (Nm; Dominant leg)	*P* = 0.30	No significant difference between *N* and PSD for any values of isometric or isokinetic strength.
	30 ± 6 yrs, 1.62 ± 0.06 m, 67 ± 5.0 kg	Retiring: 03:00 h: m Rising: 05:30 h: m	PSD		MVCext and MVCflex of knee (rad s¯¹)	*P* = 0.80	No significant difference between *N* and PSD for MVC, only a time-of day difference observec.
Blumert et al. (2007)	9 collegiate national-caliber male weightlifters	Retiring: 24:00 h: m Rising: 08:00 h: m	N	1RM	Snatch 1RM (kg)	*P* > 0.05	No significant differences between *N* and sleep loss for all performance variables.
	20.7 ± 1.2 yrs, 178.6 ± 7.8 cm, 102.3 ± 28.1 kg (1 yr°)		24 h sleep loss		Snatch VL (kg)	*P* > 0.05	
					Snatch TI (kg)	*P* > 0.05	
					Clean and jerk 1 RM (kg)	*P* > 0.05	
					Clean and jerk VL (kg)	*P* > 0.05	
					Clean and jerk TI (kg)	*P* > 0.05	
					Front squat 1RM (kg)	*P* > 0.05	
					Front squat VL (kg)	*P* > 0.05	
					Front squat TI (kg)	*P* > 0.05	
Brotherton et al. (2019)	15 resistance-trained males	Retiring: 23:00 h: m Rising: 06:30 h: m	N	Handgrip Strength (Takei Kiki Kogyo, Tokyo, Japan)	Grip Strength (N.m^− 1^; Dominant hand, three repetitions)	***P*** ** = 0.019**	Grip strength was significantly better in *N* and PSDN compared to PSD; *N* = 43.9 ± 3.8 N.m-1 vs. PSD = 42.5 ± 3.1 N.m-1 vs. PSDN = 43.4 ± 3.4 N.m-1.
	22.7 ± 2.5 yrs, 1.82 ± 0.08 m, 89.0 ± 13.8 kg (2 yrs°)	Retiring: 03:30 Rising: 06:30 h: min	PSD	Bench Press (MuscleLab, Ergotest version 4010, Norway)	Average Power (W)	***P*** ** < 0.0005**	Average power was significantly better in *N* and PSDN compared to PSD; *N* = 423 ± 78 W vs. PSD = 376 ± 59 W vs. PSDN = 410 ± 70 W.
		Retiring: 03:30 Rising: 06:30 h: min + 1-h nap at 13:00	PSDN		Average Force (N)	***P*** ** = 0.003**	Average Force was significantly better in *N* and PSDN compared to PSD; Average force significantly higher in *N* compared to PSDN; *N* = 567 ± 140 N vs. PSD = 548 ± 138 N vs. PSDN = 561 ± 142 N.
					Peak Velocity (m.s^− 1^)	***P*** ** < 0.0005**	Peak velocity significantly higher in *N* and PSDN compared to PSD; Peak velocity significantly higher in *N* compared to PSDN; *N* = 0.79 ± 0.19 m.s-1 vs. SD = 0.71 ± 0.16 m.s-1 vs. PSDN = 0.76 ± 0.17 m.s-1.
					Time to Peak Velocity (s)	*P* = 0.062	Time to peak velocity significantly faster in PSDN compared to PSD; PSD = 0.44 ± 0.15 s vs. PSDN = 0.41 ± 0.15s.
					Distance (cm)	*P* = 0.684	No significant difference between N, PSD and PSDN.
				Leg Press (MuscleLab, Ergotest version 4010, Norway)	Average Power (W)	***P*** ** = 0.001**	Average power was significantly better in *N* and PSDN compared to PSD; *N* = 956 ± 189 W vs. PSD = 901 ± 169 W vs. PSDN = 944 ± 156 W.
					Average Force (N)	*P* = 0.062	No significant difference between N, PSD and PSDN.
					Peak Velocity (m.s^− 1^)	*P* = 0.144	No significant difference between N, PSD and PSDN.
					Time to Peak Velocity (s)	*P* = 0.090	No significant difference between N, PSD and PSDN.
					Distance (cm)	***P*** ** = 0.032**	Distance significantly faster in PSDN. compared to PSD; PSD = 0.44 ± 0.15 s vs. PSDN = 0.41 ± 0.15s.
Chen et al. (2017)	10,125 University students Men = 6251 Women = 3874		< 7 h of sleep	Handgrip Strength (Dynamometer, TKK 5401 Takei Kiki Kogyo, Tokyo, Japan)	Grip Strength (N.m-1; both hands 2 repetitions)	***P*** ** < 0.001**	Grip strength was significantly lower in < 7 sleep duration ( 40.2 N) compared to 7–8 h (41.8 N) and > 8 h (41.4 N).
	range: 16–30yrs		7–8 h				
			> 8 h				
Gallagher et al. (2023)	15 recreational active male (1–2°)	Retiring: 02:30 h: m Rising: 06:30 h: m	PSD	Handgrip Strength (Dynamometer, Takei Kiki Kogyo, Tokyo, Japan)	Grip Strength (N.m-1; both hands 3 repetitions)	*P* = 0.211	No significant difference between on left or right hand grip strength
				1 RM	Bench press (Kg)	*P* > 0.05	No significant difference between nap and bench press
					Back Squat (Kg)	*P* > 0.05	No significant difference between nap and back squat
HajSalem et al. (2013)	21 male judokas	Retiring: 22:30 − 23:30 h: m Rising: 06:00 h: m	N	Handgrip strength (Calibrated hand dynamometer, T.K.K. 5401; Takei, Tokyo, Japan)	Grip Strength (N.m-1)	*P* > 0.05	Hand grip performance was significantly decreased before 5 min judo match to after the judo match. No significant difference between PSDE and N
	19.1 ± 1.2 yrs, 176.5 ± 4.2 cm, 77.3 ± 6.3 kg	Retiring: 22:30 h: m Rising: 03:00 h: m	PSDE	Wingate test (Friction-loaded cycle-ergometer; Monark 894E, Stockholm, Sweden)	PP (W.Kg¯¹)	***P*** ** < 0.01**	PP was significantly decreased before 5 min judo match to after the judo match during *N* and PSDE (*p* < 0.01). However, PP and MP was significantly decreased after PSDE when compared with *N* (*p* < 0.05).
					MP (W.Kg¯¹)		MP was significantly decreased from before 5 min judo match to after the judo match during *N* and PSDE (*p* < 0.01). However, PP and MP was significantly decreased after PSDE when compared with *N* (*p* < 0.05).
Kujawa et al. (2020)	67 physical education students Men = 29 Women = 38		N	Muscle strength of knee (UPR-02 A/S chair with Moment II by Sumer software)	Right knee extensor strength (kg)	***P*** ** = 0.21**	Right knee extensor strength was significantly decreased when compared to control group, *n* (F = 17.74, *P* < 0.001, η²*p* = 0.21).
	21.52 ± 1.58 yrs, 23.43 ± 2.34 kg/m²		24 h sleep deprivation		Left knee extensor strength (kg)	***P*** ** = 0.09**	Left knee extensor strength was significantly decreased only in females when compared with control group (F = 6.61, *P* = 0.012, η²*p* = 0.09).
Legg and Patton (1987)	25 experienced artillery soldiers		N	Grip strength (Hand dynamometer)	Isometric grip strength (right hand; Kg)	*P* > 0.05	No significant difference between *N* and PSD.
						***P*** ** < 0.05**	Grip strength was significantly lower on day 3 of the trial for *N* and PSD.
			PSD	Wingate test ( Bodyguard cycle ergometer- modified with a lever arm for instantaneous application of resistance)	Upper body PP (W. kg ¯¹)	*P* > 0.05	No significant difference between *N* and PSD.
					MP (W. kg ¯¹)	*P* < 0.01	Upper MP was significantly decreased in PSD when compared to control group (7.3%).
					Power decrease (W. sec ¯¹)		
				Anaerobic power	Lower body PP (W. kg ¯¹)	*P* < 0.001	Lower body PP was significantly increased in PSD when compared to control group (14.7%).
					MP (W. kg ¯¹)	*P* < 0.001	Lower body MP was significantly increased in PSD when compared to control group (17.0%).
					Power decrease (W. sec ¯¹)	*P* < 0.05	Lower body Power decrease was significantly increased in PSD when compared to control group (18.1%).
Meney et al. (2009)	11 healthy males	Retiring: 22:30 h: m -23:30 h: m Rising 05:00 h: m	N	Grip Strength (Dynamometer, Takei Kiki Kogyo, Tokyo).	Left grip strength	*P* = 0.10	No significant difference between *N* and SD.
	24.54 ± 2.33 yrs, 73.09 ± 5.6 kg		SD		Right grip strength	*P* > 0.05*	No significant difference between *N* and SD.
				Portable dynamometer (Takei Kiki Kogyo, Tokyo)	Leg strength	*P* > 0.05*	No significant difference between *N* and SD.
					Back strength	***P*** ** = 0.01**	No significant difference between *N* and SD.
Reilly et al. (1994)	8 male subjects		N		Biceps curl (Kg)	*P* > 0.05	No significant difference between SD and control group.
	18–24 yrs	Retiring: 02:45 h: m Rising: 05:45 − 06:00 h: m	PSD		Bench press (Kg)	***P*** ** < 0.001**	Bench press showed significant difference between normal and sleep deprived conditions only on day 3.
				Leg press machine	Leg press (Kg)	***P*** ** < 0.001**	Leg press was significantly decreased between normal and sleep deprived conditions only on day 3.
					Dead lift (Kg)	***P*** ** < 0.001**	Dead lift was significantly decreased between normal and sleep deprived.
Skein et al. (2010)	10 male team-sport athletes		N	Intermittent sprint (Motorized treadmill - True 825 SDFT System; ETL Testing Laboratories, Inc. Cortland, NY)	Mean sprint time (s)	*P* = 0.01	Mean sprint times were significantly slower during night without sleep on day 2 compared to day 1 (*P* = 0.04). During the 11 to 20 min, 31 to 40 min, and 41 to 50 min phases, mean sprint times were slower in night without sleep on day 2 than day 1 (*P* = 0.03 - *P* = 0.05).
	21 ± 3 yrs, 81.5 ± 9.5 kg, 178.6 ± 9.2 cm		Night without sleep				
				MVC (Cycle ergometer − 828E; Monark, Stockholm, Sweden)	Peak voluntary force	***P*** ** = 0.02**	Peak volunatary force was significantly decreased in night without sleep on day 1 when compared with *N* on day 1.
					Voluntary activation	*P* = 0.05	Voluntary activation was significantly reduced in night without sleep on day 2 compared with *N* on day 2
Souissi et al. (2013)	12 male judokas	Retiring: 22:30 h: m Rising: 06:00 h: m	N	Handgrip (Calibrated hand dynamometer - T.K.K. 5401; Takei, Tokyo, Japan).	Maximal hangrip strength (dominant) (Kg)	***P*** ** < 0.001**	Handgrip strength was significantly lower the evening before and after randori in SDE (∼ 3.1–8.4%), compared to *N* (*P* < 0.001).
	18.6 ± 2.4 yrs, 177.75 ±5.79 cm, 77.09 ±10.74 kg (10 yrs°)	Retiring: 03:00 h: m Rising: 06:00 h: m	SDB	MVC	Elbow flexor muscles (N)	***P*** ** < 0.001**	MVC significantly decreased (∼ 15–24%) only during SDE in the afternoon before and after the judo combat, compared to *N* (*P* < 0.001).
		Retiring: 23:00 h: m Rising: 02:00 h: m	SDE	Muscle power - Wingate test (Friction - loaded cycle ergometer (Monark 894E; Stockholm, Sweden)	PP (W. kg ¯¹)	***P*** ** < 0.001**	PP significantly decreased (∼ 3.9–9.0% ) in the afternoon before (*P* < 0.01) and after the combat (*P* < 0.001) in the SDE condition when compared to N.
					MP (W. kg ¯¹)	***P*** ** < 0.001**	MP significantly decreased (2.6–6.6%) in the afternoon before (*P* < 0.01) and after the combat (*P* < 0.001), in the SDE condition when compared to N.
Vaara et al. (2018)	20 male cadets		60-h SD	Maximal aerobic performance (Bicycle ergometer test-Ergoline, Ergometrics, 800s, Germany)			
	26 ± 2 yrs, 1.77 ± 0.01 m, 79.6 ± 11.1 kg			Maximal strength (Specifically constructed chair with a strain gauge)	Maximal isometric force (N)	*P* > 0.05	No significant changes in PRE SD (778 ± 166) and post SD (754 ± 162 N)
					Maximal rate of force development (RFD)	*P* > 0.05	No significant changes after SD (PRE & POST SD)

M = Morning, LM = Late morning, A = Afternoon, E = Evening, h = hours, m = metre, km = kilometre, min = minute, s = seconds, yrs = years, kg = kilogram, W = Watts, Hz = Hertz, BMI = Kg/m², MTB = mountain bike, n/a = not available, * = *P* value not specified in manuscript, ° = Experience, PSDE = Partial sleep deprivation at the end of the night, N = Normal Sleep, SD = Sleep deprivation, PSDN = Partial Sleep Deprivation with 1-h Nap, T0 = before, T1 = after, PP = Peak power, MP = Mean power, PSD = Partial sleep deprivation, TDC = Total distance covered, MVC = Maximal voluntary isometric contraction, SDB = Partially deprived in the beginning, SDE = Partially deprived at the end of the night

*Statistical significance (*P* < 0.05) is indicated in **bold**

### Study characteristics

The detailed participant characteristics are shown in Table 1. A total of 10,346 participants, of which 6426 were males (62.1%) and 3920 were female (37.9%), were included across the 13 studies. The number of participants per study ranged from a total of 8 to 10,125 participants, and the average number of participants per study was 796 or a median of 15 participants per study. Five studies (38.5%) focused on partial sleep loss and another five studies (38.5%) focused on complete SD. Three studies (22.5%) incorporated a 24-hour sleep loss of subjects, and one study (7.7%) underwent 60-hour of sleep loss.

Retiring times during normal conditions ranged from 22:30-hour to 24:00-hour, while rising times ranged from 05:00 h to 08:00 h, as reported by 6 studies, with minimum amount of sleep 6-hour to a maximum of 8-hour sleep. SD studies consisted of athletes either retiring to bed at “normal times” in the evening (between 22:30-hour − 23:00-hour) and rising early in the morning (between 02:00 h– 03:00 h) in two studies [19, 20] or retiring late in the morning (between 02:30-hour 03:30-hour) and rising at “normal times” in the morning (between 05:30-hour − 06:30-hour) in five studies [2, 5, 16, 20, 21]. The total amount of sleep during the one-night SD ranged from 2.5-hour to 4-hour. Only one of the studies combined both forms of SD [20], while one study did not report any sleeping times [22].

Seven studies performed handgrip strength as a strength measure, of which two studies focused only on the dominant hand [20, 21], three studies on both left and right hand [4, 5, 23], one study just on the right hand [22], and one study did not mention the protocol [19]. A total of five studies tested for maximal isometric knee contraction/leg strength [2, 4, 16, 21, 24] using an isokinetic dynamometer, a portable dynamometer or a leg press machine, with one study assessing elbow flexor muscle strength [20], and one study assessing back strength, bicep curl and bench press [16]. Two studies performed 1 repetition max (1RM) on strength measures such as snatch, clean and jerk, front squat, back squat and/or bench press [5, 15], while one study assessed measures of average power, average force, peak velocity, time to peak velocity and distance using a force-velocity linear encoder for bench press and leg press, respectively [21]. A further four studies assessed muscle strength using running [25] and/or cycling as the performance modality [19, 22, 25, 26].

Measures of handgrip strength significantly reduced in three studies following PSD, or reduced sleep. However, four studies did report that there was no statistically significant difference in grip strength between normal sleep *versus* PSD [4, 5, 19, 22]. Several measures related to bench press, leg press, back strength and dead lift also showed values to be significantly reduced following SD [21]. However, several studies found that bicep curl, leg strength [4],, 1RM bench press, 1RM back squat [5], measures related to snatch, clean and jerk, and front squat [15] were no different following SD. Two out of three studies assessing mean and peak power reported a drop due to SD [19, 22] while only one reported no significant difference [20]. Finally, maximal isometric, isokinetic knee strength and maximal voluntary contraction of the knee were not affected by a night of SD in a study performed by Bambaeichi et al. [2]. However, a study performed by Kuwaja et al. [24] found both the left and right knee extensor muscles to be significantly decreased following a period of 24-hour SD.

### Methodological quality control and publication bias

The ROBINS-I tool was used in 7 non-randomised studies, with the detailed results provided in Fig. 2. All studies showed a low risk of bias due to selection of participants (Domain 2), due to deviations from intended interventions (Domain 4), due to missing data (Domain 5), and in selection of the reported results (Domain 7). Three studies showed moderate bias due to confounding [9, 22, 23], while only one study showed moderate bias in classification of interventions [23]. All studies except for one displayed moderate bias in measurement outcomes [2]. Bambaeichi et al. [2] was the only study that showed low bias in all domains. Overall, 4 studies have a low overall risk of bias judgement, and 3 studies had a moderate overall risk of bias judgement.

The Risk of Bias (ROB) 2.0 tool was used in 6 studies, with the detailed results provided in Fig. 2. All studies showed a low risk of bias due to missing outcome data (Domain 3) and bias in the measurement of the outcome (Domain 4). One study showed some concern in bias arising from the randomization process [26], and one study showed some concern in bias in the selection of the reported result [15]. All studies showed some concern in bias due to deviations from intended intervention. Overall, 4 studies have a low overall risk of bias judgement, and 2 studies had some concern in overall risk of bias judgement.

## Discussion

The present systematic review analysed data from 13 studies that compared the effects of sleep loss or SD across one night or over extended nights on muscle strength measures. The main findings of this review were: (1) most research papers (*N* = 10; 76.9%) established significant differences in at least one of the assessed strength measures between sleep loss conditions vs. normal sleep; (2) various sleep loss protocols affect strength measures differently and to varying degrees.

### Grip strength

Grip strength has been investigated in seven studies. Grip strength was found to be significantly reduced in three studies. Grip strength was better in normal and PSD with 1-hour nap compared to SD (dominant hand); 3 h of PSD repeated over two nights lead to a decrease in maximal grip strength in the SD (2.7%) compared to the normal condition [21]. Bench press and hand grip values both improved to levels comparable to normal sleep after a 1-hour powernap. Because weightlifting is done in the evening rather than the morning, the homeostatic component increases due to the increased time awake. Grip strength was significantly lower after 7 h of sleep compared to 7–8 h and more than 8 h [23]. IGF-1 has been linked to better sleep. SD causes a rapid decrease in IGF-1 levels [27]. Hand grip performance was significantly lower before and after a 5-minute judo match [19], and it was significantly lower the evening before and after randori in SD at the end of the night (3.1–8.4%) compared to ormal sleep [20]. The anaerobic system is important in judo combat as it provides short, quick bursts of maximum power and strength [28]. As a result, lactate accumulation and exhaustion of energetic reserves caused by repeated short-term maximal efforts with incomplete recovery may decrease peak power, mean power, and handgrip after the judo match [22]. Grip strength was significantly lower on day 3 of the trial for normal sleep and PSD. This could be due to decreased muscle fiber recruitment or a change in motor unit firing frequency [22]. Only one study found no significant difference between normal and SD handgrip strength for the right and left hand [4]. Since a causal link between temperature and muscle strength is thought to exist, the minimum level at 02:00 in the SD condition could be related to an equivalent drop in temperature in this situation [29].

### Maximal voluntary contractions (MVC)

MVC was investigated in five papers, according to the findings. Three studies found significant decrease, MVC was significantly decreased only during PSD at the end of the night in the afternoon before and after the judo combat, compared to normal sleep [20]. These benefits could be attributed to increased core temperatures and stronger aerobic engagement in energy production during afternoon exercise [30]. The central and peripheral processes may change during the day; maximal voluntary force was much lower on the first night without sleep compared to the first day of normal sleep ( [25]. When compared to the control group, right and left knee extensor strength were significantly reduced, although left knee extensor strength was significantly reduced only in females [24]. This study’s findings show that the effects of SD on muscles differ between extensor and flexor muscles, and between men and women. Changes may be influenced by limb dominance. Extensor muscle strength changes are significant in children who have a dominant left leg. Changes in the strength of the right lower limb extensor muscles because of SD may also be linked to lower limb dominance. Also, during the menstrual cycle, females’ muscle recovery was found to be delayed. It is possible that female hormones and menstrual cycles play a role in the association between sleep duration and muscle strength in females [23]. According to Lanshammar et al. [31], women exhibit significant asymmetry of lower limb muscular strength while flexing the non-dominant limb and extending the dominant lower limb. In this group of individuals, the dominant lower limb’s muscles were 8.6% weaker in knee flexors but 5.3% greater in knee extensors than the non-dominant lower limb’s muscles. Bambaeichi et al. [2] found no significant difference between normal sleep and partial sleep loss for maximum voluntary knee extensor and flexor contraction, whereas after the control and partial sleep-loss nights, peak torque of knee flexors was significantly higher (5.9%) in the evening compared to the morning. After the control and partial sleep-loss nights, changes in body temperature may be associated with higher peak torque in the afternoon than in the morning [32, 33]. However, Vaara et al. [26] found no significant differences.

### 1RM

Previous night’s sleep duration did not affect the 1RM weights for the snatch, clean and jerk, and front squat, as reported by Blumert et al. [15]. The study suggested that a 24-hour period of SD might be insufficient to induce the anticipated performance decline, given the inclusion of several demanding motor tasks. Similarly, Reilly et al. [16] found that one night of PSD had no impact on maximal lifting performance in exercises such as the biceps curl, bench press, leg press, and deadlift. Meney et al. [4] also observed no significant differences in muscle strength, including right and left grip strength, leg strength, and back strength, following one night of total sleep loss. Overall, the evidence indicates that 1RM strength is generally resistant to short-term SD, suggesting that complex motor tasks are not significantly affected by brief sleep loss.

### Strength and limitations

This systematic review has several strengths, including adherence to the PRISMA 2020 guidelines and the use of Cochrane guidelines for inclusion criteria, ensuring methodological rigor and transparency. The comprehensive search strategy across multiple databases and grey literature, combined with independent and blinded screening by multiple reviewers, minimized selection bias. This systematic review has several limitations. First, there is significant variation in sample sizes across studies, ranging from 8 to 10,125 participants, which affects the robustness and generalizability of the findings. We reported both the mean and median participant numbers and acknowledged the limited statistical power due to the small sample sizes in most studies. Second, there is heterogeneity in testing methodologies, including differences in strength assessments and testing timing following SD, complicating direct comparisons. Future studies with larger, more homogeneous cohorts and standardized protocols are needed to confirm the effects of SD on muscle strength performance.

## Practical application and conclusion

Practical takeaways from this review highlight the importance of adequate sleep for maintaining muscle strength and athletic performance. Athletes and coaches should prioritize sleep as part of training and recovery strategies, recognizing that the impact of SD varies by muscle group, exercise type, gender, and limb dominance. This variation underscores the need for individualized training and recovery plans. Coaches may need to adjust training intensity following sleep loss to prevent injury and optimize performance. Additionally, healthcare providers should educate athletes about the importance of sleep and consider intervention strategies, such as sleep hygiene education and relaxation techniques, to improve sleep quality and mitigate the negative effects on muscle strength.

While the findings of this review suggest that SD may negatively impact muscle strength, especially as indicated in handgrip strength and MVC measures, the small number of participants in individual studies and the limited number of studies showing statistically significant effects restrict the generalizability of these conclusions. These limitations highlight the need for more robust, well-powered studies to confirm these trends. Future research should also explore the underlying mechanisms of sleep-related neuromuscular changes and evaluate targeted interventions to reduce the impact of sleep loss on muscle strength.

## Electronic supplementary material

Below is the link to the electronic supplementary material.

Supplementary Material 1

## Data Availability

This manuscript has no associated data.

## Abbreviations

SD: Sleep deprivation
PSD: Partial sleep deprivation
